# Multiattribute utility assessment of outcomes of treatment for head and neck cancer.

**DOI:** 10.1038/bjc.1997.158

**Published:** 1997

**Authors:** S. C. Hodder, M. J. Edwards, M. R. Brickley, J. P. Shepherd

**Affiliations:** Department of Oral Surgery, Medicine, and Pathology, University of Wales College of Medicine, Dental School, Cardiff, UK.

## Abstract

Good clinical practice is dependent on continuous audit. Most audits of head and neck cancer treatment planning have been subjective, with only 5-year survival rates being considered objectively. Improvements in clinical care require not only measurable goals that relate to patients' perspectives, but also a means of assessing to what extent those goals have been met. In this context, 5-year survival rates are too crude to be useful, although they remain important for other reasons. Because a simple clinical objective measure of outcome applicable to head and neck cancer is not available, multiattribute assessment techniques were used to develop a clinically based scale for outcomes following treatment for head and neck cancer, with domains centred on social function, pain, physical appearance, eating and speech problems, nausea, donor site problems and shoulder function. Domains were weighted relative to each other; pain (mean weight 85) and social function (89) were considered most important followed by physical appearance (76), eating (76) and speech problems (74) A series of graded statements was constructed within each domain and scaled relative to each other. These components were also combined into an overall scale that will enable objective outcome assessment in this important area of medical care.


British Journal of Cancer (1997) 75(6), 898–902
© 1997 Cancer Research Campaign

### Multiattribute utility assessment of outcomes of treatment for head and neck cancer

**SC Hodder, MJ Edwards, MR Brickley and JP Shepherd**

Clinical Decisions Research Group, Department of Oral Surgery, Medicine and Pathology, University of Wales College of Medicine, Dental School, Cardiff, UK

**Summary** Good clinical practice is dependent on continuous audit. Most audits of head and neck cancer treatment planning have been subjective, with only 5-year survival rates being considered objectively. Improvements in clinical care require not only measurable goals that relate to patients' perspectives, but also a means of assessing to what extent those goals have been met. In this context, 5-year survival rates are too crude to be useful, although they remain important for other reasons. Because a simple clinical objective measure of outcome applicable to head and neck cancer is not available, multiattribute assessment techniques were used to develop a clinically based scale for outcomes following treatment for head and neck cancer, with domains centred on social function, pain, physical appearance, eating and speech problems, nausea, donor site problems and shoulder function. Domains were weighted relative to each other; pain (mean weight 85) and social function (89) were considered most important followed by physical appearance (76), eating (76) and speech problems (74) A series of graded statements was constructed within each domain and scaled relative to each other. These components were also combined into an overall scale that will enable objective outcome assessment in this important area of medical care.

**Keywords:** health status; outcome assessment (health care); head and neck neoplasms

Four-fifths of mouth cancers are squamous cell carcinomas. The clinical presentations of various head and neck tumours have been well documented and the International Classification of Diseases (World Health Organization, 1978) provides a coding classification for malignant neoplasms arising within the oral cavity and adjacent structures. There are two main modalities of head and neck treatment, radiotherapy and surgery, used singularly or in conjunction with each other.

Over the years, a range of multidimensional quality of life (QOL) instruments have been developed to assess patients' physical, psychological and social functioning in head and neck cancer. These measures vary in their level of application. There are non-specific instruments designed for use across a wide range of chronic disease populations, such as the Sickness Impact Profile (Bergner et al, 1981), the Nottingham Health Profile (Hunt et al, 1981) and the Karnofsky Index Scale (Karnofsky et al, 1948), and there are specific measures designed for more homogeneous patient populations. The Head and Neck Questionnaire (UW QAL) describes important daily living dysfunction or limitations that patients complain of as part of head and neck cancer effects (Sammay et al, 1993); and the European Organization for Research and Treatment of Cancer Core Quality of Life Questionnaire for Head and Neck patients (EORTC QOL-H&N37) includes 37 items concerning disease and treatment-related symptoms, social function and sexuality (Bjordal et al, 1994). However, despite the importance of assessing outcome of treatment, a simple clinically relevant scale has not been developed, partly because the best and worst health states at the extremities of such a scale are not obvious. An overall measure of health outcome in this context must include social, economic, physical and psychological aspects of health (Selby et al, 1984). Although well-validated scales exist to assess psychological health in isolation (Telfer and Shepherd, 1993), assessment of the physical and functional outcomes of treatment, together with the social effects of these, has traditionally been highly subjective. It has been shown that patients find it difficult to combine such multidimensional problems into a single measure. However multiattribute assessment techniques exist (Carter, 1992; Kent, 1992) which facilitate this process. Much has been written on the use of such measures for breast cancer, gastrointestinal and testicular cancer, but little on head and neck cancer, and nothing in relation to intra-oral cancer (Stalpers, 1989).

*Received 12 March 1996*
*Revised 17 September 1996*
*Accepted 4 October 1996*

*Correspondence to:* M R Brickley, Clinical Decisions Research Group, Department of Oral Surgery, Medicine and Pathology, University of Wales College of Medicine, Dental School, Heath Park, Cardiff CF4 4XY, UK

Components that should be included may be classified into four main areas:

(1) Physical and occupational function, for example energy and ability to carry out expected normal activity.
(2) Somatic problems, for example pain, nausea, vomiting and other symptoms that result from illness or treatment.
(3) Psychological state.
(4) Effect on social interaction and ability to make contact with others.

If a scale is to be useful then it should grade outcomes with both accuracy and reliability. Assessment of psychological function and social function is difficult but multiattribute techniques can facilitate the incorporation of such subjective values into a clinically useful scale (Schipper et al, 1984).

There is a discrepancy between patients' and clinicians' perceptions of outcomes in the management of oral cancer. Patients'

objectives for treatment may be unrealistic and differ from their perceptions of actual treatment outcome, even when, in a technical sense, treatment is successful. It has been stated in other contexts that the definition of success differs widely between patients and their doctors. In treatment planning, the clinician should seek to assess the patients' needs and desires and develop a strategy that will meet their needs rather than formulate stereotyped treatment plans. This process is difficult however, and where objective measures of outcome health are not available it may be impossible.

A practical scale should be quick and easy to complete. It is also important that the scale demonstrates validity and consistency between measurements. A scale should be sufficiently sensitive to distinguish degrees of dysfunction, especially as this is a major criticism of the use of 5-year survival rates.

An accepted method of quantifying preference is developing and attaching utility values to each possible outcome following a clinical decision (Vertinsky and Wong, 1975). Utility in its strictest sense has been based on a scale with extremes of total health and death. Utilities in relation to head and neck cancer are not, however, easily quantified using this approach. Traditionally, utility assessment techniques also require that respondents integrate all components of health into a single value. There is evidence that people have difficulty in doing this (Schipper et al, 1984).

It has become increasingly apparent that the factors that influence patients' perceptions are not only medical but also social, economic and cultural (so-called 'domains' in utility parlance). While traditional utility assessment methods, such as standard gamble and time trade-offs, attempt to assess medical utility, they fail to integrate these other factors (Keeney and Raiffa, 1976). Multiattribute assessment (MAU) methods, on the other hand, explicitly allow considerations of non-medical domains and have therefore been adopted for the current project. These methods have been well validated by Boyle and Torrance (1984).

The current study therefore develops a composite outcome utility measure for head and neck cancer treatment using multiattribute techniques investigated by expert clinicians. It also facilitates the production of a new scale, designed for clinical use and based on a sound statistical framework.

### MATERIALS AND METHODS

The scale was developed and tested in three phases:

### Phase 1

Patients who had undergone treatment for head and neck cancer (35) and consultant and maxillofacial surgeons (5) were interviewed by a trained research worker to identify critical contributing factors of overall health status . The interviews were unstructured and included enquiry about physical, functional and social dimensions of health. Following this, interview transcripts were made and interview abstraction techniques as described by Babbie (1992) were used to determine the critical contributing factors of health following treatment for head and neck cancer. This process was carried out by a delphi panel to ensure that the domains did not reflect the analyst's own opinion. This panel consisted of a head and neck oncologist, four maxillofacial surgeons who regularly treat head and neck tumours, a head and neck oncology/counsellor and a researcher familiar with decision-making techniques. The delphi panel considered the interview transcripts and each member first produced a list of factors that he or she felt were important contributions to the outcome health state without reference to other members. These were then discussed with the group, who reached consensus on a list of factors. The individual factors were further consolidated by the group to yield eight final domains, each representing a significant component of health. A series of statements were derived for each domain, which described a range of outcome states from the best possible to the worst possible outcome ('intradomain statements') – for example, a domain centred on pain included statements of 'no pain' and 'pain requiring regular analgesia with opiates.'

### Phase 2

After the domains and intradomain statements had been derived, ten head and neck surgeons were asked to weight the eight domains relative to one another using a standardized visual analogue scale (interdomain weights). Anchor points for the scale ranged from 'unimportant' to 'extremely important' (Figure 1).

The same respondents were then asked to assign scores to each of the intradomain statements within each domain using the 'feeling thermometer' method as described by Boyle and Torrance (1984). The statements were weighted relative to one another on a visual analogue scale (Figure 1), thus allowing a numerical value to be assigned to each statement ('intradomain scores'). Both intradomain scores and interdomain weights by this group were subjected to statistical analysis to produce mean values, standard deviations and minimum and maximum values.

### Phase 3

The inter- and intradomain weightings derived in phase 2 were used to construct a utility scale for outcomes of head and neck cancer treatment by application of the following equation:

$$U = \frac{\Sigma(W_j S_j)}{\text{Total}} \times 100 \qquad \text{(i)}$$

where $j$ = each domain in turn; $W_j$ = interdomain weighting for domain; $S_j$ = score for a particular outcome in relation to domain; $U$ = multiattribute utility; and Total = maximum possible sum of $W_j S_j$ (value = 54502).

This resulted in a multiattribute utility score varying between zero and 100, with zero representing the worst possible health state and 100 the best possible health state.

Please mark a cross on the line to indicate how important each factor is to you in your treatment planning of patients with oral carcinoma.

*Social function,*

Unimportant |——————————————x—| Extremely important

(89 mm)

**Figure 1** Visual analogue scale

© Cancer Research Campaign 1997

**Table 1** Domains and interdomain weighting scores

| Domains | Mean | Standard deviation | Range | |
|---|---|---|---|---|
| | | | Minimum | Maximum |
| 1. Social function | 89 | 6 | 82 | 98 |
| 2. Pain | 85 | 13 | 64 | 98 |
| 3. Physical appearance | 77 | 27 | 5 | 100 |
| 4. Eating problems (swallowing) | 76 | 16 | 47 | 95 |
| 5. Speech problems | 74 | 19 | 44 | 100 |
| 6. Nausea | 69 | 17 | 43 | 95 |
| 7. Donor-site problems | 46 | 17 | 18 | 67 |
| 8. Shoulder function (stiffness/drop) | 46 | 24 | 13 | 86 |

Scores are shown to the nearest integer.

### RESULTS

Domains identified as being important during phase 1 are described in Table 1 together with mean interdomain weightings, range and standard deviations for each, based on the respondents' weightings (phase 2). Each domain represents an important component of overall health status following treatment for head and neck cancer. The domains of pain (mean weight 85) and social function (89) were considered to be the most important aspects of health, closely followed by physical appearance (77), eating problems (76) and speech problems (74) by the expert respondents in this study.

The individual intradomain statements derived by the delphi panel for each domain are shown in Appendix 2, while the mean scores (on a 100-point scale) and standard deviation data are shown in Table 2 (derived in phase 2). These data together with those presented in Table 1 can be used to produce an overall utility scale for any outcome using eqn (i). A worked example is shown in Appendix 1.

### DISCUSSION

Approximately 7.6 (Office of Population Censuses and Surveys, 1992) new cases of head and neck cancer per annum per 100 000 of UK population are detected, and the majority of these (60%) are within the oral cavity. Despite the importance of this disease, no objective measures of outcome have been developed to date and, indeed, treatments have been assessed in subjective terms by an ad hoc combination of the surgeon's perceived value of the outcome and his or her perception of the patient's opinion of the outcome. The research described here has developed a scale for an objective assessment of outcome of head and neck cancer management, has rated the relative importance of the components of this assessment and has structured them in such a way that they can be used in a questionnaire format. The scale presented here will be useful in many contexts, including audit studies, to evaluate the relative merit of varying treatment modalities, to examine to what extent actual treatment outcome is related to patients' perceptions of the value of outcome and to indicate to patients preoperatively the likely outcome of a proposed treatment plan. It will be important to validate this scale further. This should include comparison with existing scales (caution should be exercised, however, as they are all either too non-specific or measure different aspects of outcome) and also with patient–professional assessments of outcome for a series of cases. Further work is in progress to achieve this.

The scale developed here was rated using the opinions of oral and maxillofacial surgeons trained in the treatment of head and neck cancer. Clearly, the opinion of such professionals is important in the overall process of valuing outcomes of treatment. However, further studies are needed to investigate the values that patients may place on outcomes in this area. Such research would highlight important differences between the perceptions of clinicians and their patients.

Interestingly, in relation to the perceived relative importance of the components of health following treatment, surgeons agreed on the importance of social function but there was a wide range of weight values for other components. This suggests that this group of skilled clinicians attached differing importance to the various components of outcome and, therefore, that it is possible that this affects their treatment decisions. This lack of consensus deserves further research. Nevertheless, this new scale is useful in comparing the outcomes of this critical clinical activity.

**Table 2** Oral oncology outcome scale

| Intradomain statement | Social function | | Pain | | Physical appearance | | Eating problems | | Speech problems | | Nausea | | Donor site problems | | Shoulder function | |
|---|---|---|---|---|---|---|---|---|---|---|---|---|---|---|---|---|
| | Mean score | s.d. | Mean score | s.d. | Mean score | s.d. | Mean score | s.d. | Mean score | s.d. | Mean score | s.d. | Mean score | s.d. | Mean score | s.d. |
| 1 (Best) | 97 | 3 | 94 | 6 | 98 | 3 | 97 | 3 | 98 | 3 | 97 | 3 | 98 | 3 | 98 | 3 |
| 2 | 56 | 24 | 78 | 10 | 89 | 5 | 59 | 19 | 85 | 8 | 67 | 21 | 79 | 9 | 76 | 13 |
| 3 | 34 | 13 | 57 | 17 | 52 | 17 | 32 | 12 | 5 | 19 | 41 | 16 | 63 | 12 | 56 | 16 |
| 4 | 14 | 6 | 37 | 16 | 28 | 11 | 15 | 10 | 3 | 14 | 21 | 8 | 28 | 13 | 33 | 12 |
| 5 (Worst) | 5 | 5 | 13 | 10 | 9 | 5 | 14 | 28 | 6 | 6 | 6 | 5 | 10 | 9 | 11 | 11 |

Scores are shown to the nearest integer. See Appendix 2 for details of the specific statements relating the outcome levels (1 = best, 5 = worst) for each domain.

 *© Cancer Research Campaign 1997*

### REFERENCES

Babbie E (1992) *The Practice of Social Research*. Wadsworth Publishing Company: Belmont, CA

Bergner M, Bobbit RA and Carter WB (1981) The Sickness Impact Profile: conceptual formation and methodology for the development of a health status measure. *Int J Health Serv* **6**: 393–415

Bjordal K, Ahlner-Elmqvist M, Tollesson E, Jensen AB, Razavi D, Maher EJ and Kassa S (1994) Development of a European Organisation for Research and Treatment of Cancer (EORTC) questionnaire module to be used in quality of life assessments in head and neck cancer patients. *Acta Oncol* **33**: 879–885

Boyle MH and Torrance GW (1984). Developing multiattribute health indexes. *Med Care* **22**: 1045–1057

Carter WB (1992) Psychology and decision making: modelling health behaviour with multiattribute utility theory. *J Dent Educ* **56**: 800–807

Hunt SM, McKenna SP and Williams J (1981) Reliability of a population survey tool measuring perceived health problems: a study of patients with osteo-arthritis. *J Epidemiol Commun Health* **35**: 297–300

Karnofsky AD, Abelmann WH, Craver LF and Burchenal JH (1948) The use of the nitrogen mustards in the palliative treatment of carcinoma. *Cancer* **1**: 634–656

Keeney RL and Raiffa H (1976) *Decisions with Multiple Objectives*: Preferences and Value Trade Offs. Wiley: New York

Kent DL (1992) The basics of decision analysis. *J Dent Educ* **56**: 791–799

Office of Population Censuses and Surveys (1988) *Cancer Statistics Registrations (1992)*. HMSO: London

Sammy J, Hassan MD and Weymuller EA (1993) Assessment of quality of life in head and neck cancer patients. *Head and Neck* **15**: 485–496

Schipper H, Clinch J, McMurray A and Levitt M (1984) Measuring the quality of life of cancer patients: the functional living index-cancer: development and validation. *J Clin Oncol* **2**: 472–483

Selby PJ, Chapman Jaw, Etazadi-Amoli J, Dalley D and Boyd NF (1984) The development of a method for assessing the quality of life of cancer patients. *Br J Cancer* **50**: 13–22

Stalpers LJA, Verbeek ALM and Van Daal WAJ (1989) Radiography or surgery for $T_2N_0M_0$ glottic carcinoma? A decision analytic approach. *Radiother Oncol* **14**: 209–217

Telfer MR and Shepherd JP (1993) Psychological distress in patients attending an oncology clinic after definitive treatment for maxillofacial malignant neoplasia. *Int J Oral Maxillofac Surg* **22**: 347–349

Vertinsky I and Wong E (1975) Eliciting preferences and the construction of indifference maps: a comparative evaluation of two measurements methods. *Socio-economic Planning Sciences* **9**, 15–24

World Health Organisation (1978) *International Classification of Diseases, 9th revision, Basic Tabulation List with Alphabetical Index*. HMSO: London

### APPENDIX 1 A WORDED EXAMPLE OF PRACTICAL APPLICATION

A patient has undergone surgical treatment for head and neck cancer. She has slight facial deformity that is noticeable to the clinician only and her shoulder function is normal. She suffers from intermittent nausea once or twice a week, which is not managed with medication. Following the procedure, there was some wound breakdown at the donor site. She suffers occasional pain but this can be relieved with mild analgesics such as paracetamol. She has been able to return to work 1 month after surgery, has no speech difficulties and is able to eat normally. She has the following scores in each domain.

| Domain | Description fitting patient | Intradomain scores (from Table 2) |
|---|---|---|
| Shoulder function | No problems | 98 |
| Speech problems | Speech normal to trained ear | 98 |
| Social function | Able to work | 97 |
| Eating problems | Able to eat a normal diet | 97 |
| Physical appearance | Slight deformity noticeable to clinician only | 89 |
| Donor site problems | Partial graft/wound breakdown | 79 |
| Pain | Pain requiring intermittent mild analgesics | 78 |
| Nausea | Nausea once or twice a week | 67 |

The overall utility (U) for this patient would then be:

$$U = \frac{(\text{intradomain score for physical appearance} \times \text{interdomain weight for physical appearance}) + \text{etc}}{\text{maximum score (54502) using eqn (i)}} \times 100$$

$$U = \frac{(89 \times 77) + (79 \times 46) + (67 \times 69) + (78 \times 85) + (98 \times 46) + (97 \times 89) + (98 \times 74) + (97 \times 76)}{54502} \times 100$$

$$U = \frac{6853 + 3634 + 4623 + 6630 + 4508 + 8633 + 7252 + 7372}{54502} \times 100$$

$$U = \frac{49505}{54502} \times 100$$

$$U = 90.8$$

Therefore, on a scale of zero (worst possible health) to 100 (best possible health), this patient would have an overall utility of 90.8.

© Cancer Research Campaign 1997

### APPENDIX 2 INTRADOMAIN STATEMENTS FOR EACH DOMAIN

### 1. Social function

1. Life back to normal (working)
2. Social life limited to friends and family
3. Socializes with family
4. Rarely goes out
5. Housebound.

### 2. Pain

1. No pain
2. Pain requiring intermittent mild analgesics (paracetamol)
3. Pain requiring regular analgesics (paracetamol or non-steroidal)
4. Pain requiring regular non-steroidal or intermittent synthetic opiates
5. Pain requiring regular opiates.

### 3. Physical appearance

1. No visible deformity
2. Slight deformity noticeable to the clinician only
3. Deformity noticeable to the family and friends
4. Obvious facial deformity
5. Severe facial deformity.

### 4. Eating problems (swallowing)

1. Patient able to eat normal diet
2. Patient able to eat soft foods
3. Patient on liquidized diet
4. Patient on fluids only
5. No diet by mouth if possible (enteral feeding required).

### 5. Speech problems

1. Speech normal to trained ear
2. Speech problems to trained ear only (sh sounds, etc.)
3. Speech defects to untrained ear
4. Patient has to repeat some words
5. Speech mainly unintelligible.

### 6. Nausea

1. No nausea
2. Nausea once or twice a week
3. Nausea every day, requires antiemetic on a regular basis
4. Nausea for which second line antiemetics are required (ondansetron)
5. Nausea not controlled with oral medication, continuous infusion required.

### 7. Donor site problems

1. No problems
2. Partial graft/wound breakdown
3. Pain limitation of function, up to 4 weeks' treatment needed for healing
4. Residual deformity with limitation of function
5. Patient has long-term donor site dysfunction/symptoms.

### 8. Shoulder function (stiffness/drop)

1. No problems
2. Pain on full rotator cuff movement
3. Pain on normal movement
4. Limited movement because of pain
5. No movement without pain.

© Cancer Research Campaign 1997


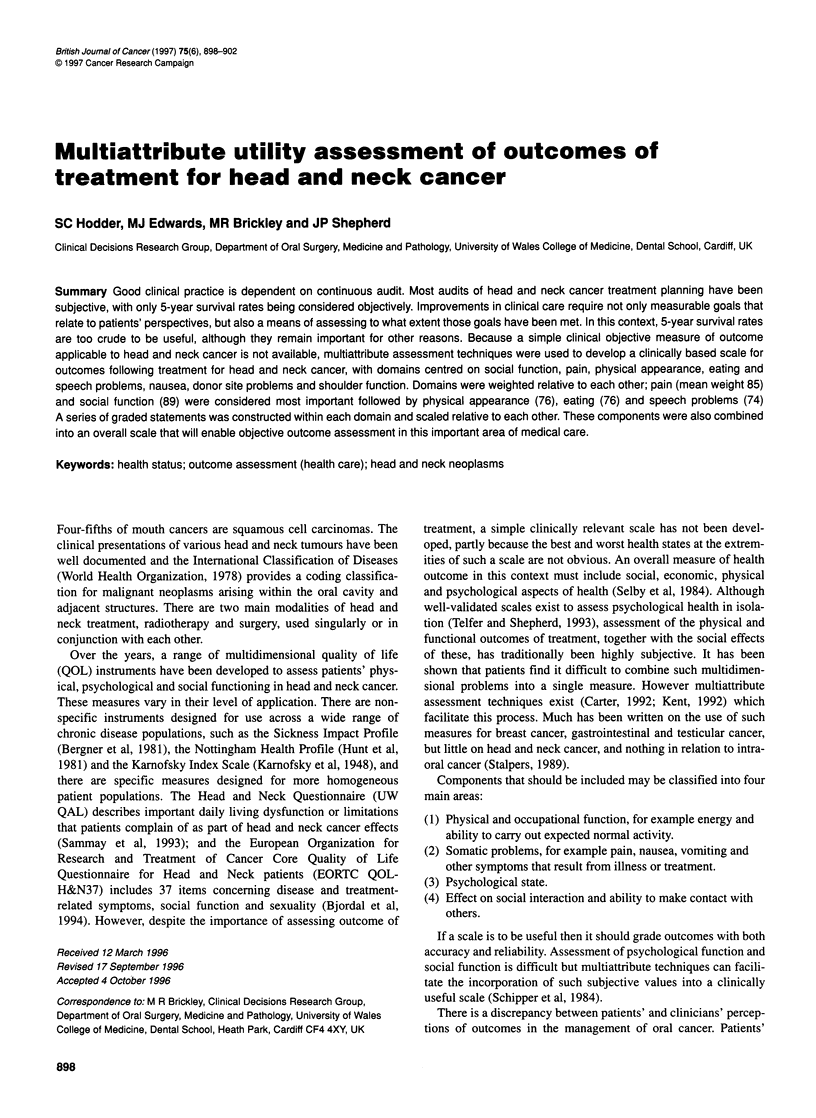

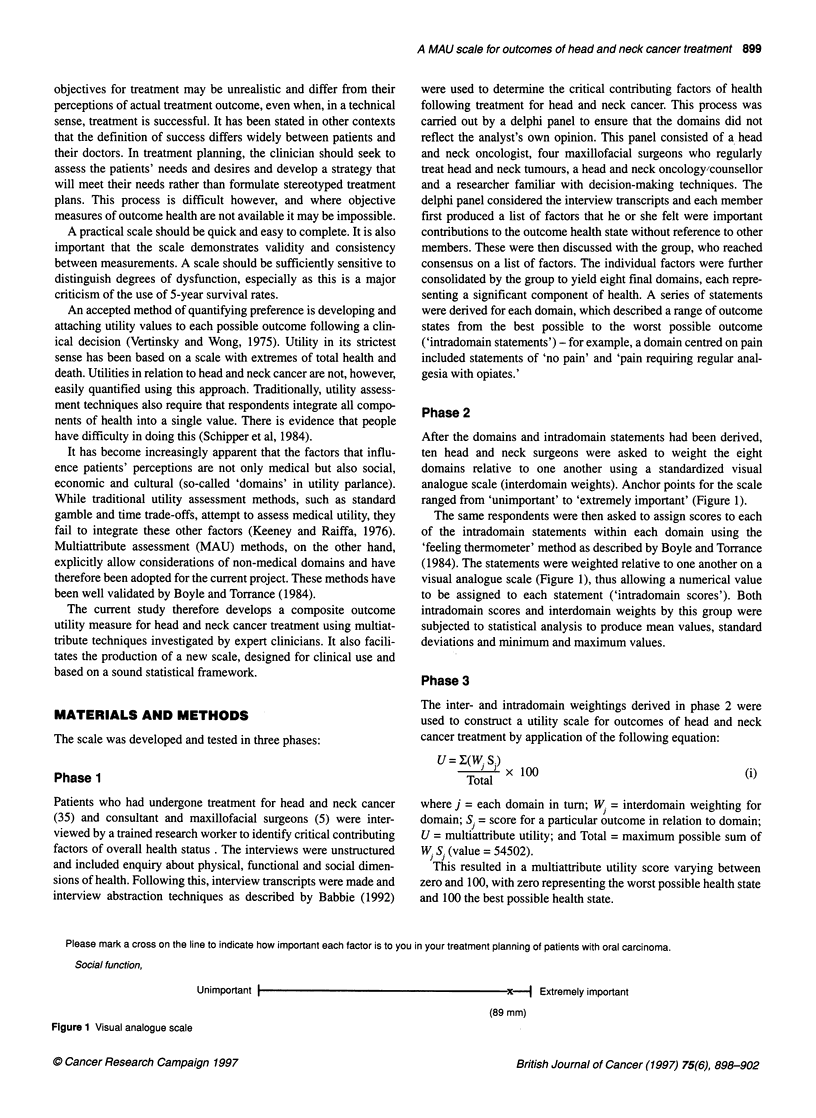

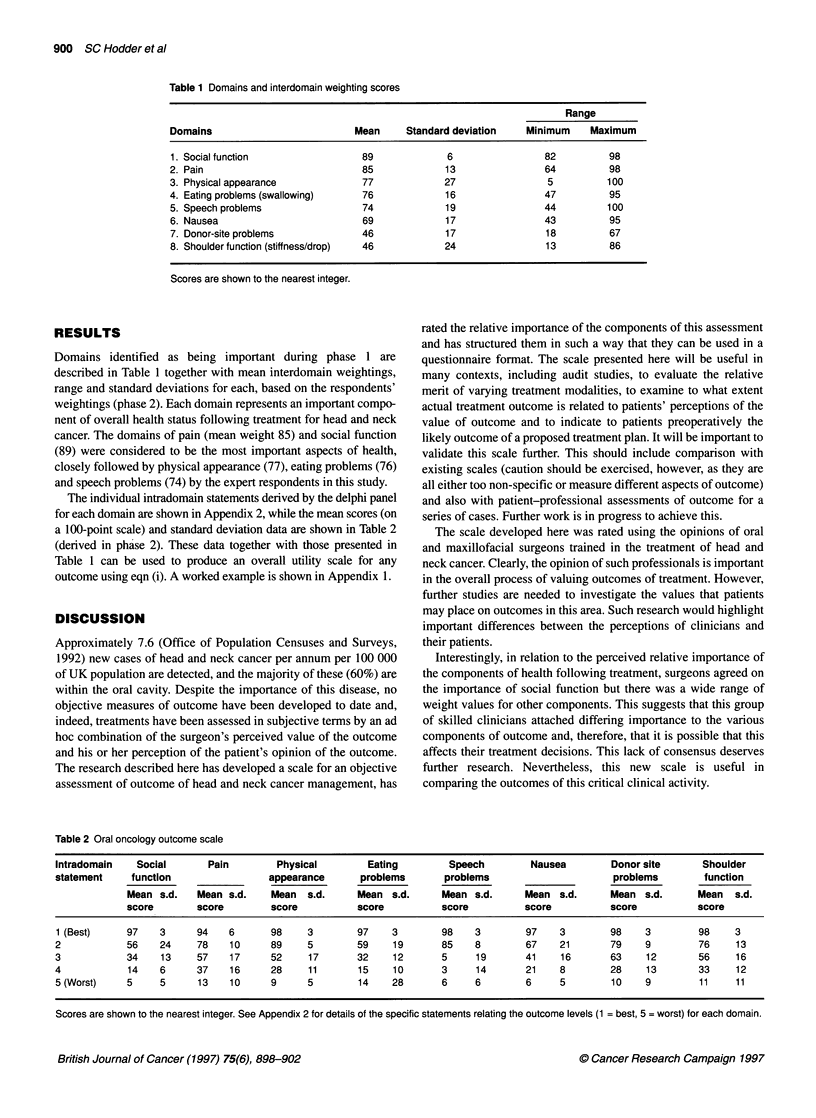

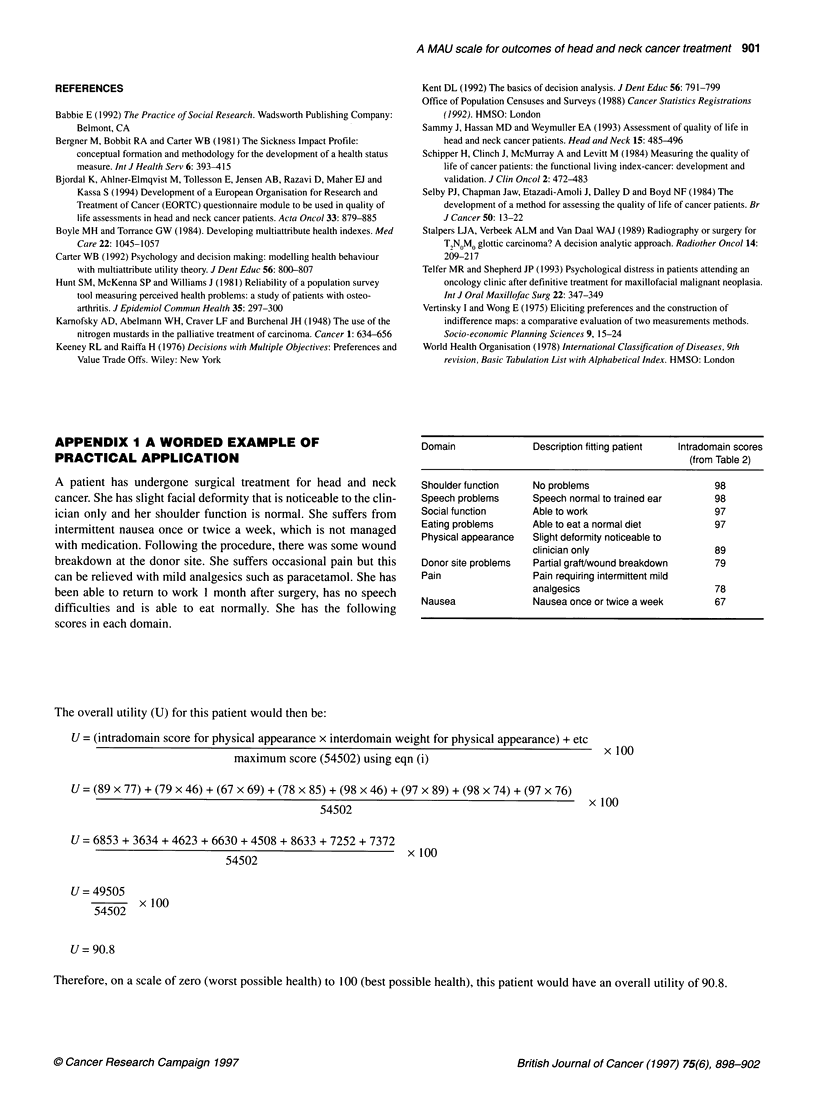

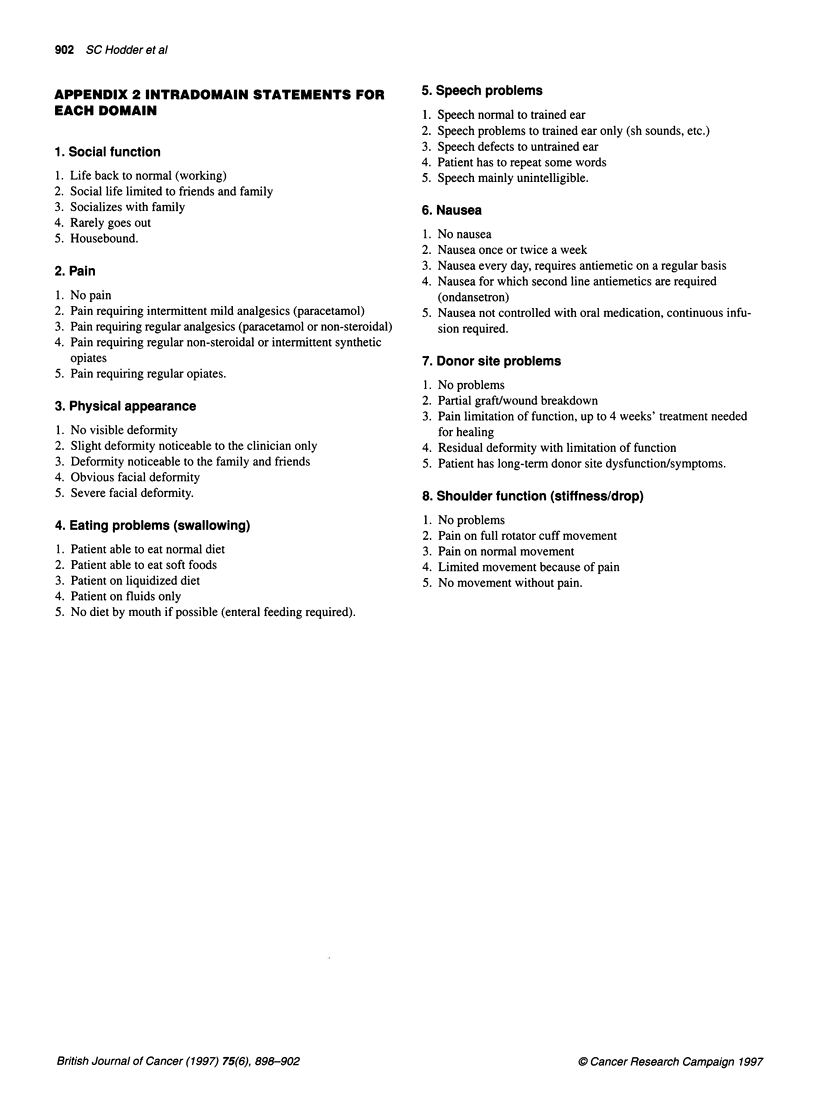


## References

[OCR_00357] Bjordal K., Ahlner-Elmqvist M., Tollesson E., Jensen A. B., Razavi D., Maher E. J., Kaasa S. (1994). Development of a European Organization for Research and Treatment of Cancer (EORTC) questionnaire module to be used in quality of life assessments in head and neck cancer patients. EORTC Quality of Life Study Group.. Acta Oncol.

[OCR_00364] Boyle M. H., Torrance G. W. (1984). Developing multiattribute health indexes.. Med Care.

[OCR_00368] Carter W. B. (1992). Psychology and decision making: modelling health behavior with multiattribute utility theory.. J Dent Educ.

[OCR_00391] Hassan S. J., Weymuller E. A. (1993). Assessment of quality of life in head and neck cancer patients.. Head Neck.

[OCR_00372] Hunt S. M., McKenna S. P., Williams J. (1981). Reliability of a population survey tool for measuring perceived health problems: a study of patients with osteoarthrosis.. J Epidemiol Community Health.

[OCR_00395] Schipper H., Clinch J., McMurray A., Levitt M. (1984). Measuring the quality of life of cancer patients: the Functional Living Index-Cancer: development and validation.. J Clin Oncol.

[OCR_00400] Selby P. J., Chapman J. A., Etazadi-Amoli J., Dalley D., Boyd N. F. (1984). The development of a method for assessing the quality of life of cancer patients.. Br J Cancer.

[OCR_00405] Stalpers L. J., Verbeek A. L., van Daal W. A. (1989). Radiotherapy or surgery for T2N0M0 glottic carcinoma? A decision-analytic approach.. Radiother Oncol.

[OCR_00410] Telfer M. R., Shepherd J. P. (1993). Psychological distress in patients attending an oncology clinic after definitive treatment for maxillofacial malignant neoplasia.. Int J Oral Maxillofac Surg.

